# Improving allocative efficiency from network consolidation: a solution for the health workforce shortage

**DOI:** 10.1186/s12960-022-00732-1

**Published:** 2022-07-15

**Authors:** Theepakorn Jithitikulchai

**Affiliations:** 1 Faculty of Economics, Thammasat University, 2 Prachan Road, Phranakorn, Bangkok, 10200 Thailand; 2 Takemi Program in International Health, Harvard T.H. Chan School of Public Health, 677 Huntington Ave, Boston, MA 02115 United States of America; 3 World Bank Group, 1818 H Street, NW, Washington, DC 20433 United States of America

**Keywords:** Health workforce, Health resources, Resource allocation, Health catchment area, Community health planning, Community health network

## Abstract

**Background:**

Public hospitals are facing a critical shortage of health workers. The area-based network consolidations could be the solution to increase the system capacity for human resources by improving local allocative efficiency.

**Methods:**

This study develops counterfactual simulations for area-based network allocations for the health workforce in 10500 public hospitals in Thailand and examines improvements in allocative efficiency from the health workforce redistribution at different administrative levels such as sub-districts, districts, provinces, and health service areas. The workload per worker is calculated from the output measured by numbers of outpatient and inpatient cases and the input measured by numbers of health workers. Both output and input are weighted with their economic values and controlled for heterogeneity through regression analysis. Finally, this study compares the workload per worker and economic valuation of the area-based networks or ex-ante scenarios with the hospital-level or status quo scenario.

**Results:**

Network consolidations of the sub-district primary-level hospitals within the same district could reduce workload per worker by seven percentage points. Another practical policy option is to consolidate similar hospital levels such as primary, first-level secondary, and mid-level secondary hospitals altogether within the same province which could result in the reduction of the workload per worker by 6–7 percentage points. The total economic value gained from consolidating similar hospital levels within the same province is about 15–18 percentage points of total labor cost in the primary hospitals.

**Conclusion:**

This study illustrates the improvement in allocative efficiency of the health workforce in public hospitals from the area-based network consolidations. The results provide an insightful example of economic gains from efficiently reallocating the medical workforce within the same local areas. Major reforms are required such that the health care delivery units can automate their resources in corresponding to the population's health needs through a strengthening gatekeeping system.

**Supplementary Information:**

The online version contains supplementary material available at 10.1186/s12960-022-00732-1.

## Introduction

An important goal of human resource planning in a health system is to settle an adequate health workforce with balanced allocation in any specific administrative areas [1]. The major challenges of health resource allocation in Thailand are the scarcity of health workforce and the inequitable access to quality health care [2]. The country is facing the problem of higher demand for health services that exceeds the available capacity of the public health system [3].

Even though the geographical allocations of health workers in Thailand have been improved significantly over the last three decades with higher workforce availability, the number of health workers is still lower than the official requirement, and the public hospitals under the Office of the Permanent Secretary in the Ministry of Public Health (MOPH) still have chronic shortages in the health workforce. In addition to the insufficient budget [4], there are geographical allocation issues such that the health workforce is more concentrated in Bangkok and big cities and the inequity gap in proportions of the targeted population to medical doctors could reach almost ten times differential among provinces [5].

In particular, the shortage of nurses has been a critical issue for the Thai public health system, and the problem could be more severe [6, 7]. Unfortunately, the nurse resignations are quite consecutively high due to the fact that the health system cannot retain skilled and experienced nurses, not because of the inadequate production of nurses [8]. A study of 19 912 registered nurses revealed that 10 percent of the surveyed sample would like to quit their career within the next 2 years [9]. Another study [10] found that the young and less-skilled nurses have a stronger intention of resignation for having less time off, less job satisfaction, and higher stress. Other studies reported similar conclusions from community hospitals [11], a tertiary care hospital [12], and a university hospital [13].

In the future, Thailand will be facing higher demand for the health workforce in primary [14] and tertiary [15] hospital levels. The projections of demand and supply for medical specialists demonstrated severe shortages in almost all specialized medical professions as the consequences of the aged society [16]. Therefore, it is an urgency for the government to manage health workforce in order to improve allocative efficiency and achieve the desirable population health objectives.

**Fig. 1:**
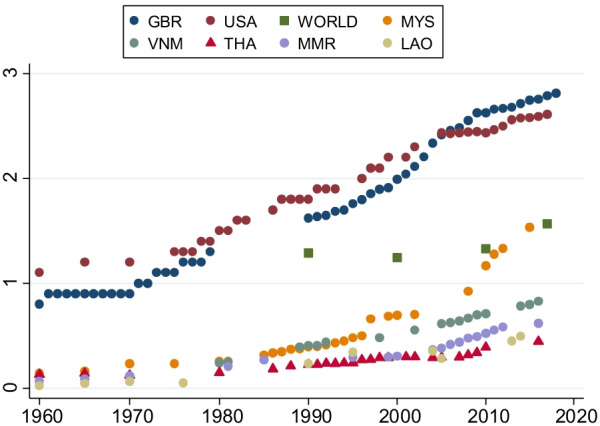
Medical doctors per 1000 people, 1960–2018. *GBR* Great Britain, *USA* United States of America, *WORLD* global average, *MYS* Malaysia, *VNM* Vietnam, *THA* Thailand, *MMR* Myanmar, *LAO* Laos (source: World Development Indicators, The World Bank)

**Fig. 2:**
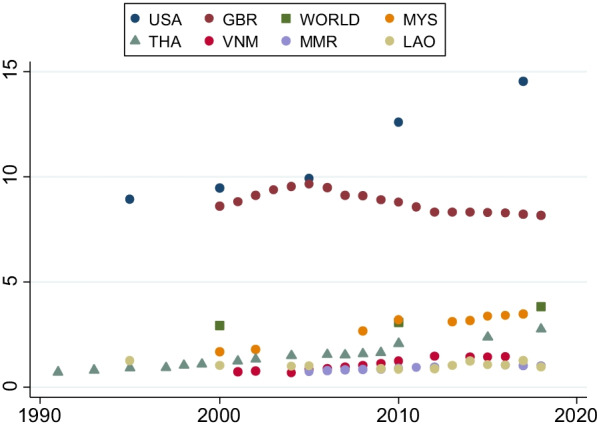
Nurses and midwives per 1000 people, 1991–2018. *USA* United States of America, *GBR* Great Britain, *WORLD* global average, *MYS* Malaysia, *THA* Thailand, *VNM* Vietnam, *MMR* Myanmar, *LAO* Laos (source: World Development Indicators, The World Bank)

Comparing with the other countries, Thailand has limited availability of health workforce. Appendix Table 7 and Figs. 1, 2 show that the Thai medical workforce per 1000 people is much lower than the developed countries or the selected countries in Asia. Appendix Table 8 illustrates that the workforce shortage in the MOPH hospitals is critical but seems to be mitigable if there is an improvement in allocative efficiency due to variations in the shortage severity at different hospital levels.

Successful health resource planning requires not only the balance in both quantitative and qualitative goals of the health workforce management, but also the adaptation to varying health system needs. Certainly, the effective reallocation for the supply of medical workers in accordance with the demand for health care will lead to the more desirable clinical outcomes [17].

This study reports efficiency gains and associated economic values from the area-based human resource allocation of hospitals under the Office of the Permanent Secretary, the MOPH. The area-based network of the health workforce in this study is a simulation application of consolidating the public hospitals within local administrative areas. It is a counterfactual quantitative exercise to measure the hospital outputs per worker and subsequent results of reduction in workload per worker from network allocation scenarios. The network consolidation should simultaneously mitigate the health workforce shortage and enhance allocative efficiency of the health workers within their local areas from improvements in the workload per worker. Due to the health workforce shortage in public hospitals in Thailand, the lower workload per worker implies increase in workforce sufficiency rather than decrease in efficiency of the health system.

In fact, the area-based network of the health workforce is not a new concept for Thailand. It has been developed and implemented by the health system and medical staffs to collaborate in the primary service of the district health administration systems for many years [18–21]. Jithitiikulchai [22] studied the area-based network consolidations for the health workforce in the MOPH hospitals. The author [22] found that the shortage situation is severe, and that the shortage could be mitigated from network reallocations. However, the analysis in the study [22] was considered by each medical profession such as doctor, nurse, dentist, pharmacist, and others. Thus, this pioneered work did not consider the aggregated output of health care service delivery units relative to the total health workforce, but only investigated by medical profession based on the number of health workforce relative to the minimum manpower requirement.

Therefore, this study endeavors to quantify whether and how the area-based resource allocation at different levels of hospital services and administrative areas could mitigate the health workforce shortage in terms of per capita workload reductions and proposes a general framework for the area-based network consolidation simulations. Specifically, this study develops the counterfactual simulation model to compare the workload per worker between (a) the hospital-level averages (status quo) and (b) the area-based network averages after consolidations at different levels of hospital services within the local administrative areas (ex ante). This approach is an application of the gatekeeping concept to optimally allocate resources according to the demand and supply of health care services to mitigate the shortage problem of the health workforce.

The methodology is straightforward, duplicable, and scalable. Simple linear regression approach is used to estimate the weights of output and input reflecting their economic values. The status quo and ex ante scenario comparisons use basic arithmetic operations. The simple economic valuation could help to recommend consolidation options that provide higher monetary values. This analysis could be an aspiring example of network consolidations for other countries facing a shortage in health workforce.

## Methods

This study develops the counterfactual network simulation exercises for the area-based health workforce allocation at different levels of hospital services within levels of local health administrative areas. The objective is to measure allocative efficiency from redistributing the health workforce to improve health system’s capacity.

The network consolidation approach is considered an application of the gatekeeping system to manage health system resources in corresponding to the demand for health care services and the workforce supply capacity within each of the local system networks. This study assumes an efficient gatekeeping system such that local health systems could automate the seamless referral system for the outpatient (OP) and inpatient (IP) patients and, accordingly, allocate the area-based workforce to minimize the shortage of the workers.

The counterfactual simulation for network consolidation analysis provides comparisons of the “workload per worker” between (a) the hospital-level averages (status quo) and (b) the network averages after consolidations at different levels of hospitals and administrative-areas (ex-ante). See Additional file 1: Figure S1 for an illustrative example of local network by the four hospitals in the same area.

The geographical administrative area levels in this study follow the national health system which are the sub-district, district, province, and health service area levels. There are five hospital levels which are primary, first-level secondary, mid-level secondary, high-level secondary, and tertiary.

This study considers network consolidations within local administrative areas with different hospital classifications:

1. All hospital levels altogether
2. Only within the same hospital level
3. Similar hospital levels:
  3.1. Type A: {Primary, First-level Secondary} and {Mid-level Secondary, High-level Secondary, Tertiary}
  3.2. Type B: {Primary, First-level Secondary, Mid-level Secondary} and {High-level Secondary, Tertiary}.

*Measurement of workload per worker*

The workload per worker is the output divided by input, in which this study calculates both output and input to reflect their costs of human resources. The weights of each outpatient and inpatient case are the relative cost of workforce assigned for each discharge. The weights of the health workforce are the weekly work hour multiplied with the hourly earnings of each health occupation. Using the relative weights implicitly assume that the relative costs of output and input could capture the differences in severity of the medical treatment cases, availability of health workforce, and intensity of the workload per worker.

*Output calculation*

The outputs of hospital or area-based network are the aggregation of the weighted OP and IP cases to reflect the relative workforce costs allocated to each discharge. The approach used to measure output in this study thus applies the case mix index (CMI) concept that provides a standard reference for the standard IP costs as the diagnosis-related group (DRG) for reimbursements from the national health insurance schemes [23].

This study calculated the average costs with the regression models separately for the five hospital levels within each of the OP or IP categories. Separating cost regression equations for different cohorts or sampling groups are common in applied econometric analysis [24–26]. Separating cost regressions provides different cost levels according to the average costs incurred by hospital level and patient category. Finally, the OP and IP cases can be weighted with the standardized costs given observable characteristics and then aggregated into the total output of each hospital or network.

The average costs of each treatment case from the regression analysis are determined by the observable characteristics such as ICD-10 principal diagnosis (PDx) codes of 140 disease categories, sex and age of patients, service time, service type, insurance type, number of days admitted (only IP treatments), and health region. The total cost of each treatment case reported from hospitals covers labor, material, capital costs, and other indirect costs. The log-linear total cost regression functions are calculated separately for OP and IP services from five different hospital levels to standardize relative weight values to each medical treatment case. For instance, medical treatments in higher-level hospitals tend to be more costly than those in the primary hospitals. Similarly, IP cases should be more expensive than OP cases.

As the cost from each treatment case is the total cost reported from hospitals, this study multiplies the predicted costs of each OP and IP case with the hospital-level share of labor cost. The share of labor cost at the hospital-level can adjust the fitted total cost of each medical case into the approximated labor cost to be used as the output weights. Thus, output is the weighted numbers of outpatient (OP) and inpatient (IP) cases.

*Input calculation*

The input factor in this study is the total number of health workers in which each medical profession is weighted with their regional averages of hourly earnings and work hours per week. Thus, the aggregation of the weighted numbers of health workers is the total workforce of hospitals or area-based networks evaluated in monetary terms to reflect economic costs of health workforce. The average work hours per week and average earning per hour are obtained from the regression models controlling for observable characteristics of the health workers in the public sector aged 15–64, using the national labor force surveys. The health professions include medical doctors, nurses, dentists, pharmacists, and other medical professions. Therefore, input is the weighted numbers of health workers.

*Area-based network allocation*

This study compares workload per worker between the hospital-level average before network consolidations (status quo) and the area-based averages after network consolidations (ex ante). The workload per worker is calculated as the ratio of the weighted aggregate output and the weighted number of workforces. The counterfactual network consolidation simulations quantify the area-based health workforce reallocation within the local administrative areas at different hospital levels. The hypothesis in this study is that the network consolidation could improve the health system efficiency by alleviating the shortage of health workforce.

This study calculates average reductions in workload per worker as the percentage differences between the averages of workload per worker from the status quo and ex ante scenarios, whereas the unit of measurements is the OP case in primary hospitals. The standardized measurement unit, using the average labor costs for OP treatments in primary health care units to calculate the number of OP cases in primary hospitals, allows comparisons of the OP and IP services across different hospital levels.

Finally, this study estimates the economic value to compare the network consolidation options across different administrative areas and hospital-level classifications, using the status quo situation as the baseline scenario. The reduction in workload per worker can be valued financially by multiplying the total workloads reduced from network consolidation with the average labor costs. The reduced number of total workloads are the multiplications of the number of health care service delivery units, average health workers per service delivery unit, average workload per worker, and the percentage reduction in workload per worker. The workload and labor costs are in units of OP cases in primary hospitals. Technical details are available in Additional file 1 for output and workforce measurements, cost regressions, weights reflecting economic valuation, and area-based network allocations.

### Data

The outpatient and inpatient discharge data from the Information and Communication Technology Center of the MOPH used in this study covers principal diagnosis (PDx), sex, age, service time (office hours or after hours), service type (walk-in, referral, among others), insurance type (Universal Coverage Scheme, Civil Servant Medical Benefit Scheme, Social Security Scheme, and others), number of days admitted (IP treatments only), and total costs of each treatment case. The OP and IP cases are the discharges in the fiscal year 2019.

Numbers of each medical profession such as the doctor, nurse, dentist, pharmacist, and other medical occupations are the hospital-level data from the Human Resource Management Division of the Office of the Permanent Secretary, the MOPH. There are 100 320 nurses, 16 593 doctors, 7906 pharmacists, and 4662 dentists who worked in the hospitals for the fiscal year 2019 as reported in Appendix Table 8.

The average hourly earnings and work hours per week of each medical profession are calculated from the quarterly Labor Force Survey (LFS) 2002Q1 to 2020Q1 of the National Statistical Office. The health workers aged 15–64 employed in the public sector are selected for each medical profession using the International Standard Classification of Occupations, ISCO-88 codes. The health workforce weights as adjustment factors are reported in Additional file 1: Table S3.

## Results

This study uses the medical case data from 10 500 public hospitals under the Office of the Permanent Secretary, MOPH, across geographical units and health regions. The output is based on 284 273 598 OP discharges and 18 971 271 IP discharges in the budget year 2019. The OP and IP cases are weighted with their estimated costs, in which the estimated OP and IP costs are controlled for observable heterogeneity through linear regression estimations separately for each of five hospitals levels. Additional file 1: Tables S4, S5 provide the regression results of the cost of OP and IP cases.

The output, the aggregations of weighted average costs of OP and IP treatments in each hospital are normalized with the average labor cost of the OP cases in primary hospitals and resulted in 1 204 133 398 normalized OP discharges in primary hospitals as the standardized output unit. There are 155 377 health workers calculated from the total workforce weighted with their regional averages of work hours per week and earnings per hour of each occupation. The output per worker is a standard unit of measurement calculated as the “OP cases in primary hospitals per worker” used in comparing the status quo and ex ante scenarios across different hospital and geographical administrative area levels. Both output and input estimates reflect the labor resources expended for medical treatments in the fiscal year 2019.

The results from the consolidation of all hospital levels altogether illustrate that the networking at the district levels can reduce the average workload per worker by about 1.8% on average or reduce from 7924 to 7785 OP cases in the primary hospitals, as shown in Table 1. Meanwhile, at the province and health region levels, the workforce consolidation could reduce by 1.5% and 1.6%. However, the networking at the sub-district level has no impact on average, such that the primary-level OP cases per worker are about the same.

**Table 1:** Area-based network allocation of all service levels

All levels	Units	Average workforce	Total normalized OP cases	Average OP cases per worker	Average reduced OP cases per worker (%)
Hospital	10 500	393	4 751 255	7924	
Sub-district	7025	396	4 770 658	7920	0.1
District	878	508	5 831 026	7785	1.8
Province	76	2616	22 294 363	7808	1.5
Health region	12	13 406	99 726 840	7801	1.6

The reduced OP cases per worker are the percentage differences of the average cases per worker after the consolidation (ex ante) compared with the average cases per worker of hospitals (status quo)

The results from the networking within the same hospital levels are illustrated in Table 2. The results show that the consolidation of workforce at the primary level and the approach of networking at the district, province, and health region levels could reduce workload per worker by 7%, 10%, and 14%, respectively. For the first-level secondary hospitals, area-based networking cannot reduce the workload. For the mid-level secondary hospitals, networking at the administrative levels of the province and health region could reduce workload per worker by 2–3%. For the high-level secondary and the tertiary hospitals, the area-based network consolidations cannot reduce the workload quantity per worker.

**Table 2:** Area-based network allocation by each service level

Each level	Units	Average workforce	Total normalized OP cases	Average OP cases per worker	Average reduced OP cases per worker (%)
1. Primary					
Hospital	9 609	3	13 709	5759	
Sub-district	6 548	5	22 807	5791	− 0.6
District	877	39	193 440	5379	6.6
Province	76	421	2 213 098	5164	10.3
Health region	12	2298	11 034 260	4929	14.4
2.1 First-level secondary					
Hospital	508	78	420 186	5510	
Sub-district	508	78	420 186	5510	0.0
District	502	79	430 050	5510	0.0
Province	66	725	4 156 579	5483	0.5
Health region	12	3120	16 650 573	5461	0.9
2.2 Mid-level secondary					
Hospital	264	117	490 123	4469	
Sub-district	264	117	490 123	4469	0.0
District	260	119	496 075	4469	0.0
Province	65	603	2 578 542	4371	2.2
Health region	12	2367	9 974 729	4329	3.1
2.3 High-level secondary					
Hospital	84	529	3 908 967	8462	
Sub-district	84	529	3 908 967	8462	0.0
District	84	529	3 908 967	8462	0.0
Province	61	710	6 172 210	8476	− 0.2
Health region	12	3073	26 335 503	8484	− 0.3
3. Tertiary					
Hospital	35	1158	17 252 208	14 037	
Sub-district	35	1158	17 252 208	14 037	0.0
District	35	1158	17 252 208	14 037	0.0
Province	34	1165	17 295 997	14 038	0.0
Health region	12	3388	39 571 635	14 068	− 0.2

The reduced OP cases per worker are the percentage differences of the average cases per worker after the consolidation (ex ante) compared with the average cases per worker of hospitals (status quo)

In an effort to network workforce in lower hospital levels, there are two options, {Primary, First-level Secondary} of Option A and {Primary, First-level Secondary, Mid-level Secondary} of Option B, as illustrated in Tables 3, 4. The results show that both options of network consolidations for similar hospital levels could reduce the average workload per worker for the lower hospital levels. When combined at the province level, Option A could reduce the workload by 6%, while Option B could reduce the workload by 7%, on average.

**Table 3:** Area-based network allocation by clustered service level (option A)

Levels	Units	Average workforce	Total normalized OP cases	Average OP cases per worker	Average reduced OP cases per worker (%)
Primary and first-level secondary					
Hospital	10 117	37	196 712	5645	
Sub-district	6803	40	211 733	5656	− 0.2
District	878	91	478 724	5421	4.0
Province	76	1071	5 972 216	5296	6.2
Health region	12	5312	27 267 885	5218	7.6
Mid-level secondary, high-level secondary, and tertiary					
Hospital	383	683	8 451 506	9744	
Sub-district	383	683	8 451 506	9744	0.0
District	376	710	9 055 009	9750	− 0.1
Province	76	1697	17 177 190	9627	1.2
Health region	12	8351	73 694 039	9527	2.2

**Table 4:** Area-based network allocation by clustered service level (option B)

Similar levels	Units	Average workforce	Total normalized OP cases	Average OP cases per worker	Average reduced OP cases per worker (%)
Primary, first-level secondary, and mid-level secondary					
Hospital	10 381	55	263 619	5373	
Sub-district	6940	58	279 078	5378	− 0.1
District	878	120	585 779	5092	5.2
Province	76	1432	7 474 182	5011	6.7
Health region	12	7364	35 889 151	4971	7.5
High-level secondary and tertiary					
Hospital	119	861	10 965 434	11 410	
Sub-district	119	861	10 965 434	11 410	0.0
District	116	896	11 757 623	11 418	− 0.1
Province	76	1260	16 639 308	11 442	− 0.3
Health region	12	6168	66 066 136	11 290	1.1

The reduced OP cases per worker are the percentage differences of the average cases per worker after the consolidation (ex ante) compared with the average cases per worker of hospitals (status quo)

However, both options could reduce the average cases per worker only by 1–2% within the health regional networks in the upper hospital levels, which are {Mid-level Secondary, High-level Secondary, Tertiary} of Option A and {High-level Secondary, Tertiary} of Option B.

*Economic valuation of network consolidation options*

The economic valuation can be compared between the status quo and ex ante scenarios. Thus, we can appraise the network consolidation options. For each network consolidation option, the aggregated reduction in a standardized unit of OP cases conducted at primary hospitals are calculated using the multiplication of the reduced primary OP cases per worker, average primary OP cases per worker, the average number of workforces in each service-delivery unit, and the total number of units. In the end, we obtain the aggregate numbers of reducible primary OP cases multiplied with the average labor cost per OP case at primary hospitals, the economic value gained from the network consolidation.

In Table 5, the total reductions in number of primary OP cases, which could be obtained from each network consolidation option comparing with the status quo scenario, are illustrated. The most reduced number in the aggregate workloads occurs from consolidating all hospital levels altogether. However, combining all hospital levels seems unrealistic and unpractical. The more reasonable options are to network similar hospital levels and combine within the provinces or health service areas.

**Table 5:** Total number of primary OP cases gained from different network consolidation levels

	All levels	Each level	Similar hospital levels	
			Option A	Option B
Sub-district	22 056 449			
District	62 457 295	12 099 738	14 689 296	26 627 800
Province	23 289 839	21 355 110	41 627 599	33 242 766
Health region	20 079 491	23 146 822	46 282 149	42 137 805

The unit of measurement is the OP case at the primary hospitals

Table 6 illustrates the economic values in Thai Baht and US dollar in correspondence with Table 5. A practical alternative with high economic outcomes is the application of combining hospitals with similar hospital levels at the provincial level. The calculation shows that, if comparing with the aggregate labor cost incurred at hospitals in the same budget year, the network consolidations of similar hospital levels within the same provinces could gain about 15–18% of total labor cost in the primary hospitals at 10 billion Thai Baht.

**Table 6:** Economic values from different network consolidation levels (in million THB and USD)

	All levels		Each level		Similar hospital levels			
					Option A		Option B	
	THB	USD	THB	USD	THB	USD	THB	USD
Sub-district	978	30						
District	2770	86	537	17	651	20	1181	37
Province	1033	32	947	29	1846	57	1474	46
Health region	890	28	1026	32	2052	64	1869	58

The THB/USD is 32.3 which is the 2011–2020 average

## Discussion

The area-based network consolidations can redistribute the health workforce and improve allocative efficiency of human resource administration. Suggested by the most practical results from the analysis in this study, networking the primary-level hospitals within the same district could reduce workload per worker by 7% on the national average. Another feasible option is the method of consolidating similar hospital levels such as primary, first-level secondary, and mid-level secondary hospitals within the same province which is estimated to reduce the workload per worker by 6–7%. Definitely, the implementation requires the strengthened primary health care units of the primary-level hospitals within each district.

Conceptually, we assume that the network consolidations occur in the situation that we have the efficient gatekeeping system to optimize resources according to the demand for health care service and the workforce supply capacity within each network. However, we should realize that the health service units are still independent of each other in planning, budgeting, and performance assessment. In addition, the current health system does not allow such flexibility to reflect in the promotion and career path for public health workers in Thailand.

Therefore, this requires what Leerapan et al. [27] proposed as “major reforms of MOPH care delivery models” such that the health care delivery units can adjust and adapt their resources and services in corresponding to the population health needs. Leerapan et al. [27]’s proposal includes the capacity reallocation of health care delivery teams to be enlarged in the areas with excess demand and to be reduced in the areas with excess supply. This proposal of “major reforms of MOPH care delivery models” is conceptually consistent with the allocative efficiency; the health system utilizes the management capacity to establish and prioritize local objectives to redistribute health system resources corresponding to the demand–supply gaps of health workforce.

Noree et al. [28] defined distinguished properties of the desirable health care delivery system as a seamless health service network of an integrated system of primary, secondary, and tertiary hospitals. Pooling resources and planning through the management information system within a local health care network are critical for a robust referral management system with the gatekeeping application. Both Noree et al. and Leerapan et al. [27, 28] aligns with the goal of the “value-based health care” concept [29, 30], which is a health care delivery model to maximize the value of care for patients and minimizing the cost of health care.

The practical possibilities in my opinion are to consolidate primary hospitals within each district, similar hospital levels within each province, or a mixture of both. Although the evaluations in this study center on the results at average, this study can provide some guidance of the policy options for optimal allocation of public health workers to mitigate workforce shortage. A good policy is not one-size-fits-all. It requires decentralization for the provincial and district health systems to have their autonomy over decision-making processes and be equipped with accountability to monitor and evaluate their performance through the health and management information systems.

The area-based networking approach at the district or provincial level could add a commuting and time burden to the health workers. This is inevitably undeniable. Therefore, we need financial incentives, career advancement mechanisms, and team development programs, among others, to facilitate the local health care system development. See [31–40] for evidence of the effective financial and non-financial incentives in Thailand and developing countries.

In addition, the gatekeeping system must consider the potentially increased travel cost burdens to the patients especially the poor living in the remote rural areas. The primary hospitals are available in every sub-district of Thailand in which those patients who can commute to their sub-district hospital should be able to access to the district-level primary healthcare network. However, the higher-level hospitals mostly locate in the city areas. The transportation services for referrals are required to support the health care accessibility of the poor and vulnerable people.

Finally, any country with community health networks should have a national strategic plan for area-based health system development that aligns with the national human resource plan. Not only the more equitable distribution is required for health workforce management across geographical and administrative areas, but also the more fiscal resources to produce the medical workforce to solve the shortage severity, as we can observe from Appendix Table 7 and Figs. 1, 2 which illustrate that Thailand has poorly low medical workforce. The author strongly encourages the health workforce organizations to call out for reprioritizing more national budget for the health system to mitigate the workforce shortage.

### Limitation

First, this study has some limitations on total output calculations. Health workforce positions have the responsibility not only on treatment service delivery used in this study. They also have some other tasks such as health promotion and disease prevention services, and administrative works, among others. Due to data limitations of the additional roles of health personnel, this study cannot consider other duties beyond the OP and IP discharges.

Second, the area-based network consolidations in this study assumed that the health workforce could move freely within the network to serve the local health care needs. However, the calculations are the technical results for the policymakers to consider policy and program options on human resource management. It requires considerate evaluations of positive and negative externalities that potentially occurred to the health workers within each hospital and the local area. The practical possibilities seem to consolidate primary hospitals within each district, similar hospital levels within each province, or combinations of both approaches. Instead of the workforce relocation, the robust referral system could assign patients to the most appropriate hospitals at the time. This could be actualized by digital transformation of the local health systems.

Third, the calculations in this study did not explicitly consider the capital inputs of hospitals. It is perhaps difficult in terms of conceptualization to incorporate the capital component into the cost regression models. Nevertheless, this study reflects the capital factor by providing a more realistic consolidation within the same or similar hospital levels to regard the capital differences between hospital levels.

Lastly, this study uses the estimated labor cost for weights of each OP and IP case. However, Porter [29, 30] suggested that achieving the goal of health care delivery requires that the value determinant should be the health outcomes achieved per every monetary unit spent. Therefore, the future research can measure the value of each discharge with the framework for performance improvement in health care that creates value for patients, measured by the outcomes achieved, not inputs nor volume of services delivered.

## Conclusion

This study evaluates shortage mitigation from the area-based network consolidations of health workers. The analytical results confirm improvement in allocative efficiency of the health workforce in the MOPH hospitals. The economic valuation reveals that consolidating similar hospital levels within the same province is an optimal solution. The benefits from efficient area-based networks are equal to 15–18% of total labor cost in the primary-level hospitals.

### Supplementary Information

**Additional file 1.** Supplementary material for methods and regression results.

## Data Availability

The datasets used for this study are available from the corresponding author upon reasonable request and with the authorized approvals from the government offices which own the original raw data.

## Appendix

See Table 7, Figs. 1, 2 and Table 8.

**Table 7:** Medical workforce per 1000 people

	Doctor	Nurse	Dentist	Pharmacist
Thailand	0.50	2.40	0.10	0.20
OECD countries				
Austria	5.16	8.10	0.57	0.69
Switzerland	4.27	17.60	0.54	0.55
Germany	4.14	13.30	0.80	0.62
ASEAN countries				
Brunei	1.50	7.80	0.23	0.12
Singapore	1.90	6.40	0.33	0.39
Malaysia	1.20	3.30	0.36	0.43

Thailand as %	Doctor (%)	Nurse (%)	Dentist (%)	Pharmacist (%)
OECD countries				
Austria	10	30	18	29
Switzerland	12	14	19	36
Germany	12	18	13	32
ASEAN countries				
Brunei	33	31	43	167
Singapore	26	38	30	51
Malaysia	42	73	28	47

Source: Health at Glance Thailand 2017. Strategy and Planning Division of Office of the Permanent Secretary, Ministry of Public Health

**Table 8:** Shortage in health workforce of the hospitals under the Office of the Permanent Secretary, MOPH. Source: Jithitikulchai [22]

	Doctor	Dentist	Pharmacist	Nurse
1. Primary				
Shortage severity index	− 2%	− 35%	26%	− 52%
Health workers	14	5	8	9826
Hospitals	3	3	3	9633
2.1 First-level secondary				
Shortage severity index	− 25%	− 19%	− 3%	6%
Health workers	3924	1866	2626	23 359
Hospitals	508	508	508	508
2.2 Mid-level secondary				
Shortage severity index	− 16%	− 17%	− 2%	2%
Health workers	2869	1231	1714	15 902
Hospitals	265	265	265	265
2.3 High-level secondary				
Shortage severity index	− 21%	0%	− 1%	4%
Health workers	4362	907	1881	25 394
Hospitals	84	84	84	84
3. Tertiary				
Shortage severity index	− 8%	− 6%	− 4%	− 19%
Health workers	5424	653	1677	25 839
Hospitals	36	36	36	36
All levels				
Shortage severity index	− 21%	− 16%	− 2%	− 47%
Health workers	16 593	4662	7906	100 320
Hospitals	896	896	896	10 526

The shortage intensity index (% of minimum manpower required) is the average of $\frac{{\left( {n_{i.j} - {\text{lb}}_{i,j} } \right)}}{{{\text{lb}}_{i,j} }} \times 100$. For the hospital $i$ and health profession $j$, the $n_{i.j}$ is the number of health worker, and the ${\text{lb}}_{i,j}$ is the minimum manpower requirements

## Abbreviations

CSMBS: Civil Servant Medical Benefit Scheme
MOPH: Thai Ministry of Public Health
PDx: Principal diagnosis
SSS: Social Security Scheme
UCS: Universal Coverage Scheme
