# Supplementary material for: Improving allocative efficiency from network consolidation: a solution for the health workforce shortage
Source: Hum Resour Health. 2022 Jul 15;20:59. doi: 10.1186/s12960-022-00732-1 (PMC9288026; doi:10.1186/s12960-022-00732-1)
Supplement: Supplementary file 1 — Additional file 1. Supplementary material for methods and regression results. [file 12960_2022_732_MOESM1_ESM.pdf]

Improving Allocative Efficiency from Network Consolidation: A Solution for the Health Workforce Shortage

SUPPLEMENTARY MATERIAL

Methods

This study endeavors to quantify the reduction of workload per worker from the area-based network allocation. The analysis conducts the counterfactual simulations to compare the workload per worker between (a) the hospital-level averages (*status quo*) and (b) the area-based network averages after consolidations at different administrative-area and hospital levels (*ex-ante*).

This study enumerates the reductions in workload per worker to evaluate economic gains from the area-based health workforce allocation policy at different levels of hospital services within the local administrative areas. This approach is an application of the gatekeeping concept to manage resources according to the demand for health care service and the workforce supply capacity within each network.

This study calculates the workload per worker from the output as the weighted numbers of outpatient (OP) and inpatient (IP) cases, divided by the input as the weighted numbers of health workers. This calculation applies to both the hospital and area-based network averages.

The output of the health system in this study is the workload which covers OP and IP services. This study applies the case mix index (CMI) approach to assign relative weights to the OP and IP cases to reflect the relative human resources allocated for each medical treatment case. The output weights are calculated from the log-linear cost regression models to

standardize the costs for human resources used in each OP or IP case. The aggregations of outputs for the hospitals or the local health system networks can be standardized with the average labor cost of the OP cases at the primary-hospital level to have the same measurement unit for comparisons across levels of administrative areas and hospitals, and OP/IP treatment categories.

The input factor is the weighted numbers of health workers, whereas the weights are the multiplications of average hourly earnings and average work hours per week. The worker or input in this study covers medical doctors, nurses, dentists, pharmacists, and other medical professions.

Conceptually, the calculations of both output and input reflect their economic values. The output weights are calculated from the observed characteristics through the cost regression models of OP and IP cases. Similarly, the calculations of the input weight components, averages of hourly earnings and weekly work hours of each medical profession, are calculated with the regression models of the health workers in the public sector.

Finally, this study compares the workload per worker from the scenarios before network consolidations (status quo) and after network consolidations (ex-ante). The counterfactual area-based network simulations are calculated for different hospital classifications: all hospital levels, only the same hospital level, and similar hospital levels. The administrative area levels in this study cover the sub-district, district, province, and health service area.

The network allocation for the health workforce considers the hospital output per worker as the baseline to evaluate the efficiency gain of human resource pooling within the area-based network. To estimate the economic value, the reductions of workload per worker can be straightforwardly calculated for the total workload reductions and then multiplied with the average labor costs. Therefore, we can compare the network consolidation options across

different administrative areas and hospital-level classifications from the economic gain differentials.

This study assumes an efficient gatekeeping system such that local health systems could distribute the OP and IP patients and, accordingly, allocate the workforce to minimize the shortage of the workers. Assuming that the health professions could be perfectly substituted is unrealistic. Nevertheless, this study endeavors to quantify the health workforce resources as the total budget allocated to the health system. Therefore, instead of making the perfect substitution assumption, this study explicitly assumes the gatekeeping system efficiency.

Output of medical service in public hospitals

The main output equation can be described as following:

$$\hat{y}_i = \widehat{OP}_i + \widehat{IP}_i$$

where $\hat{y}_i$ is the estimated quantity of medical service outputs for hospital i, which composes of $\widehat{OP}_i$ as the weighted number of outpatient cases and $\widehat{IP}_i$ as the weighted number of inpatient cases.

The weighted number of outpatient cases $\widehat{OP}_i$ is calculated from

$$\widehat{OP}_i = \hat{C}_i^{OP} Q_i^{OP} \times shr\_labor\_cost_i$$

which $\hat{C}_i^{OP}$ is a vector of standardized total costs for each outpatient discharge calculated by hospital i, Q_i^{OP} is a multipliable vector of ones for all outpatient cases in hospital i, and $shr\_labor\_cost_i$ is the hospital-level share of labor cost. Each element of Q_i^{OP} represents outpatient case which implicitly contains attributes as regressors shown in Table S1.

Table S1. Regressors for the log cost regression functions of outpatient and inpatient treatments

Variable	Description	Outpatient	Inpatient
PDX_{ICD-10}	ICD-10 Principal Diagnosis (PDx) codes of 140 disease categories	×	×
age	Age	×	×
age^2	Age squared	×	×
$female$	Sex	×	×
$intime$	Dummy variable of service time (1=office hours, 2=out-office hours)	×	×
$typein$	Dummy variables of service type (1=walk-in, 2=by appointment, 3=refer from other hospital, 4=refer from emergency service or EMS)	×	×
$instype$	Dummy variables of insurance type (1=UCS, 2=CSMBS and other state schemes, 3=SSS, 4=OOPE)	×	×
$days\_admit$	Days admitted		×
$days\_admit^2$	Days admitted squared		×
$area\_id$	Dummy variables of health regions (12 areas)	×	×

Source: OP and IP discharges in the budget year 2019, ICT Center, MOPH

Note: UCS = Universal Coverage Scheme, CSMBS = Civil Servants Medical Benefit Scheme and other relevant health insurance programs, SSS = Social Security Scheme, and OOPE = Out-of-pocket expenditure.

Therefore, the estimated $\hat{C}_{OP}$ can be calculated from the following linear regression model of the log-transformed total cost of each OP discharge:

$$\log(C_{OP}) = \alpha_0 + \sum_{j=2}^{140} \alpha_{PDX_j^{ICD-10}} \times I\left(PDX_j^{ICD-10}\right) + \alpha_{age} \times age + \alpha_{age^2} \times age^2$$

$$+ \alpha_{female} \times I(female) + \alpha_{intime} \times I(intime) + \sum_{k=2}^{4} \alpha_{typein_k} \times I(typein_k)$$

$$+ \sum_{l=2}^{4} \alpha_{instype_l} \times I(instype_l) + \sum_{m} \alpha_{area\_id_m} \times I(area\_id_m) + u$$

where $\log(C_{OP})$ is a log-transformed vector of the reported total cost for each outpatient discharge, with regressors from Table S1, where u is a vector of stochastic component independently distributed by a normal distribution with zero mean and constant variance, or $u \sim N(0, \sigma_u^2)$. The $\left(\alpha_0,\ \{\alpha_{PDX_j^{ICD-10}}\}_{j=2}^{140},\ \alpha_{age},\ \cdots\ ,\ \{\alpha_{area\_id_m}\}_m\right)$ are the outpatient cost regression parameters to be estimated. The $j,\ k,\ l,$ and m denote the subscripts for dummy variables of ICD-10 Principal Diagnosis (PDx) code, service type, insurance type, and health service area of outpatient discharges. Thus, the standardized cost controlled for observable heterogeneity in disease, patient, service, and areas: $\hat{C}_{OP}$ could be obtained from the fitted regression model.

Similarly, the weighted number of inpatient cases $\widehat{IP}_i$ is calculated from

$$\widehat{IP}_i = \hat{C}_i^{IP} Q_i^{IP} \times shr\_labor\_cost_i$$

which $\hat{C}_i^{IP}$ is a vector of standardized total costs for each inpatient discharge in hospital i, Q_i^{IP} is an all-ones multipliable vector of all inpatient cases in hospital i, and $shr\_labor\_cost_i$ is the hospital-level share of labor cost. Each element of Q_i^{IP} represents inpatient case which contains attributes as regressors in Table S1 above. Therefore, the estimated $\hat{C}_{IP}$ can be calculated from the following linear regression model of the log-transformed total cost of each IP discharge:

$$\begin{aligned}\log(C_{IP}) = \beta_0 &+ \sum_{j=2}^{140} \beta_{PDX_j^{ICD-10}} \times I\left(PDX_j^{ICD-10}\right) + \beta_{age} \times age + \beta_{age^2} \times age^2 \\ &+ \beta_{female} \times I(female) + \beta_{intime} \times I(intime) + \sum_{k=2}^{4} \beta_{typein_k} \times I(typein_k) \\ &+ \sum_{l=2}^{4} \beta_{instype_l} \times I(instype_l) + \beta_{days\_admit} \times days\_admit \\ &+ \beta_{days\_admit^2} \times days\_admit^2 + \sum\nolimits_m \beta_{area\_id_m} \times I(area\_id_m) + v\end{aligned}$$

where $\log(C_{IP})$ is a vector of log-transformed total cost reported for each inpatient discharge, with regressors from Table S1, and v is a vector of stochastic term independently distributed as $v \sim N(0, \sigma_v^2)$. The $\left(\beta_0,\ \{\beta_{PDX_j^{ICD-10}}\}_{j=2}^{140},\ \beta_{age},\ \cdots\ ,\ \{\beta_{area\_id_m}\}_m\right)$ are the inpatient cost regression parameters to be estimated. The $j,\ k,\ l$, and m denote the subscripts of dummy variables for ICD-10 Principal Diagnosis (PDx) code, service type, insurance type, and health service area of inpatient discharges. Therefore, the standardized cost for each inpatient case, $\hat{C}_{IP}$ could be obtained from the fitted regression model.

The estimations of average costs, $\hat{C}_{OP}$ and $\hat{C}_{IP}$, of medical treatment services are calculated separately by five hospital levels: primary, first-level secondary, second-level secondary, third-level secondary, and tertiary hospitals. The approach of separating cost regressions to compare means and other distributional moments is ordinary for applied econometric research. See Jones, Lomas, and Rice (2014, 2015) and Deng, Lou, and Mitsakakis (2019) as examples of separated regressions for medical costs.

The objective of this study focuses on the health workforce allocations. Thus, this study does not cover other production factors such as capital. It is arguably unpractical in terms of conceptualization to incorporate the capital component into the cost regression models. Nevertheless, this study indirectly reflects the capital factor by consolidating the hospital within the same or similar hospital levels.

Given that the unconditional mean of cost or $\bar{c}$ is approximately equal to the conditional mean predicted from the regression in numerical analysis, one can expect that the separate cost regressions provide different cost average levels according to the observed costs incurred within each hospital level. For instance, the higher-level hospitals tend to have higher cost averages than the lower-level hospitals. Similarly, this study separately standardizes the costs from OP and IP discharges, whereas the IP treatments tend to have higher average costs than

the OP treatments. This is confirmed by the estimated constant coefficients of regression results in Tables S4-S5. We also need to separate the OP and IP regression models, because the OP treatment has no duration of admission, but the number of days admitted is an important feature of the IP treatment. Variations in the cost predictions reflect the heterogeneous attributes in each medical discharge at different hospital levels and whether OP or IP categories.

The reverse transformation for the theoretically consistent predictions of the logarithmic average costs is such that

$$\hat{C} = \exp\left(\log\left(\hat{C}\right)\right) \times \exp\left(RMSE^2/2\right)$$

where $RMSE$ is the root mean squared error calculated from the differences between the standardized costs obtained by regression analysis and the reported costs from the health information system.[1]

Instead of counting each OP or IP case as the outputs for status quo and ex-ante scenarios, or equivalently assigning equal weights as ‘one’ for any discharges, this study uses the ‘relative labor cost’ weights to reflect the human resources expended for each OP and IP discharge. These weights are the estimated costs reflecting the observable characteristics of patient, service, hospital, and area for each OP or IP case. Therefore, the aggregated outputs reflect the economic values of health workforce resources used for each discharge. Tables S4-S5 at the end of this Supplementary Material report the regression results.

Reflecting labor cost component in the OP and IP costs

The estimated costs of OP and IP cases at each hospital level reflect both workloads of health workers and the other resources used. The original OP and IP costs calculated by the

[1] See theoretical discussion from pages 205-206 of Wooldridge, Jeffrey M. (2019). Introductory Econometrics: A Modern Approach. 7th edition. Cengage Learning.

Ministry of Public Health's hospitals come from the activity-based costing approach. This costing model considers both direct and indirect costs. The direct costs cover the labor, material, and capital costs, while the indirect costs are calculated as 20% of the total direct costs.

There is no information available on the labor cost of the reported OP and IP costs. Given microdata limitations, this study adjusted the estimated OP and IP costs with the hospital-level share of labor cost from the hospital financial statement in the same budget year of the OP and IP cases. This approach could help a better distinction between different workloads used in each OP or IP treatment instead of using the total costs that also cover other cost components. Thus, the quantified output in this study excludes material, capital, or another cost components. Table S2 reports the summary statistics of the share of labor cost from different hospital levels, in which this study assigns the hospital-level ratio of labor cost to each discharge.

Table S2: Summary statistics for share of labor cost

Hospital Levels	Mean	S.D.	Min	Max	N
Primary	64%	13%	26%	83%	9,609
First-level secondary	60%	7%	36%	76%	508
Mid-level secondary	55%	7%	35%	74%	264
High-level secondary	51%	7%	29%	68%	84
Tertiary	47%	6%	38%	58%	35

Source: Hospital-level trial balance sheet in the budget year 2019, Division of Health Economics and Health Security, MOPH

Workforce as inputs of public hospitals

Define $n_{i,j}$ as the number of health workforce in a public hospital i for health profession j such as medical doctor, nurse, dentist, pharmacist, and others. This study calculates relative weights for the workforce numbers of each profession by average work

hours per week and average hourly earnings. Therefore, the hospital-level or area-based aggregations of the weighted numbers of health workers are the total worker valuation in monetary terms.

This study defines N_i as the total (weighted) workforce in hospital i, which N_i is an aggregation of total numbers of medical profession j multiplied with their relative weights calculated for economic costs:

$$N_i = \sum_j n_{i,j} * \overline{hour}_{public,j} * \overline{wage}_{public,j} = \sum_j n_{i,j} * weight_j$$

where, for any profession j, $\overline{hour}_{public,j}$ is the average work hours per week, and $\overline{wage}_{public,j}$ is the average hourly earnings. The weighted number of health professionals is subsequentially in monetary term reflecting economic costs of workforce.

This study calculates the average work hours per week and average earning per hour from the National Statistical Office's Labor Force Survey 2002Q1-2020Q1. The hourly earnings are temporally and spatially adjusted by deflators calculated from the official consumer price indexes at the regional level. Both average work hours per week and average hourly earnings are estimated with the regression models controlling for heterogeneity on sex, age, education, urban/rural areas, and regions for the health workers aged 15-64 in the public sector. The estimated work hours and hourly earnings for each medical profession are the regional averages and fixed at the budget year 2019 for the same period of the OP and IP cases and the health workforce in this study. The sample sizes are too small in several provinces, so this study uses the regional representation to envisage spatial heterogeneity.

Table S3 shows the averages of $\overline{hour}_{public,j}$ and $\overline{wage}_{public,j}$ in each region. The adjustment factors in the last column are the ratios between the multiplications of average work

hours per week and average earning per hour, using the nurse profession in each region for the denominator as the base reference.

Table S3: Weights for public health workforce by profession

	$\overline{hour}_{public,j}$	$\overline{wage}_{public,j}$	$\overline{hour}_{public,j} \times \overline{wage}_{public,j}$	Adjustment Factor
**Doctor**				
Central	40.1	219.3	8,799.9	1.25
North	45.1	207.0	9,326.8	1.27
Northeast	45.3	192.9	8,743.7	1.22
South	48.1	224.6	10,809.6	1.55
**Dentist**				
Central	38.1	185.9	7,092.9	1.00
North	37.2	189.2	7,032.3	0.96
Northeast	39.2	178.1	6,976.7	0.97
South	38.9	184.2	7,173.1	1.03
**Pharmacist**				
Central	26.0	147.8	3,836.0	0.54
North	27.3	143.3	3,913.7	0.53
Northeast	27.7	139.2	3,855.6	0.54
South	27.0	152.1	4,106.7	0.59
**Nurse**				
Central	43.9	161.0	7,062.4	1.00
North	44.3	165.4	7,332.5	1.00
Northeast	45.6	157.3	7,177.9	1.00
South	44.2	158.1	6,985.8	1.00
**Others**				
Central	37.4	172.5	6,460.8	0.91
North	37.6	167.0	6,275.7	0.86
Northeast	38.6	159.2	6,151.5	0.86
South	39.2	161.1	6,309.6	0.90

Source: Labor Force Survey 2002Q1-2020Q1, National Statistical Office
Note: The estimated earnings and hours are fixed at the budget year 2019.
For each region, nurse is the base for weights of other medical professions.

Workload per worker measurement

The general concept for workload per worker is the output divided by input, which is

$$workload\ per\ worker = \frac{Output}{Input}$$

whereas the $Output$ is $\hat{y}_i = \widehat{OP}_i + \widehat{IP}_i$ which represents the weighted amount of medical treatment services delivered in the hospital i; and the $Input$, total weighted workforce used in health service delivery, is $N_i = \sum_j n_{i,j} * \overline{hour}_j * \overline{wage}_j = \sum_j n_{i,j} * weight_j$ for hospital i. Again, both output and input weights reveal economic costs in monetary terms.

Therefore, the equation for hospital workload per worker in this study is

$$workload\ per\ worker = \frac{\hat{y}_i}{N_i}$$

This analysis provides analytical results of comparing output per head before and after the area-based network consolidations at the different hospital and administrative area levels, i.e., comparing workload per worker between the status quo and ex-ante scenarios. The higher workload per worker in a public hospital does not necessarily imply higher productivity than others. However, it could exhibit the continuous problem of workforce scarcity in public hospitals.

Area-based network allocation

The counterfactual simulations of network consolidation quantify the area-based health workforce allocation within the local administrative areas. This study conducts simulations at different levels of hospital services: (a) all hospital levels altogether, (b) only within each hospital level, and (c) combining similar hospital levels. We can consider the area-based network of human resources as the gatekeeping system to optimize the system resources, given the demand for health care service and the workforce supply capacity.

The study hypothetically presumes that the network consolidations within the same administrative areas could enhance the health system's allocative efficiency by mitigating the workforce shortage. Figure S1 illustrates an example of area-based network allocation of four hospitals within the same administrative area. The existing status quo scenario postulates that the output $\hat{y}_i$ and the workers N_i are attached to only one hospital i. On the other hand, the ex-ante scenario combines the output and input from all n hospitals within the same area to optimize all feasible resources to reduce the supply- demand gap of human resources for health.

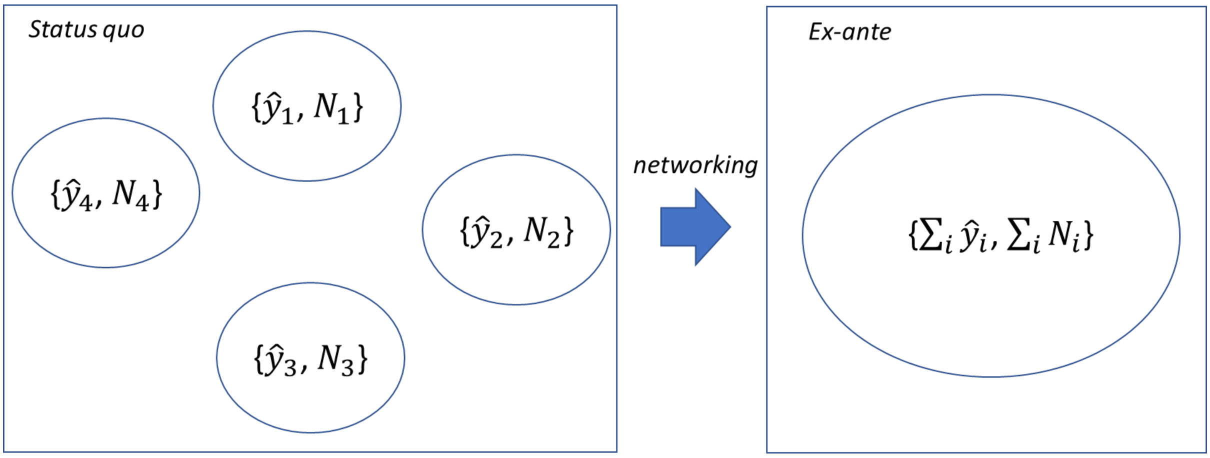

Figure S1. Example of a network of four hospitals within the same administrative area

Each hospital has the estimated quantities of $Output$ as $\hat{y}_i = \widehat{OP}_i + \widehat{IP}_i$ and $Input$ as $N_i = \sum_j n_{i,j} * \overline{hour}_{public,j} * \overline{wage}_{public,j} = \sum_j n_{i,j} * weight_j$ for hospital $i = 1,2,3,4$.

The status quo scenario of workload per worker is the average workload per worker of all four hospitals. This can be written as $\sum_{i=1}^{4} \frac{\hat{y}_i}{N_i} / 4$.

The ex-ante scenario is the average workload per worker after consolidating all four hospitals altogether. This can be written as $\frac{\sum_{i=1}^{4} \hat{y}_i}{\sum_{i=1}^{4} N_i}$.

When all the $\hat{y}_i$ are normalized into the same unit of measurements, i.e., the unit of OP case in the primary hospitals, one can compare the average reduction in workload per worker

as the percentage change between the status quo and ex-ante scenarios. For instance, it could be expressed as 1-[($\frac{\sum_{i=1}^{4}\hat{y}_i}{\sum_{i=1}^{4}N_i}$)/($\sum_{i=1}^{4}\frac{\hat{y}_i}{N_i}/4$)].

In general terminology, the status quo situation for the average workload per worker of n hospitals within a local administrative area can be expressed as the following:

$$\sum_{i=1}^{n}\frac{\frac{\hat{y}_i}{N_i}}{n}$$

On the other hand, the ex-ante situation for the average workload per worker after combining the output and input from all n hospitals within the area can be expressed as the following:

$$\frac{\sum_{i=1}^{n}\hat{y}_i}{\sum_{i=1}^{n}N_i}$$

Therefore, the average reduction in workload per worker of this area can expressed in a general form as the following:

$$1-\frac{\frac{\sum_{i=1}^{n}\hat{y}_i}{\sum_{i=1}^{n}N_i}}{\sum_{i=1}^{n}\frac{\hat{y}_i}{N_i}/n}$$

At the aggregated levels of administrative areas of interested, such as health service areas $m = 1,2,\cdots$, the average reduction in workload per worker from consolidating within each of the health service areas can be expressed as the following:

$$1-\frac{\sum_m\frac{\frac{\sum_{i_m=1}^{n_m}\hat{y}_{i_m}}{\sum_{i_m=1}^{n_m}N_{i_m}}}{m}}{\sum_{\substack{i_m=1\\ \forall m}}^{n_m}\frac{\hat{y}_{i_m}}{N_{i_m}}}$$

for i_m and n_m denoted the hospital i_m with a total of n_m hospitals in the health service area m. The nominator is the average *ex-ante* workload per worker across areas, while the denominator is simply the average *status quo* workload per worker of all hospitals from every health service area. Essentially, this formula is the comparison of the status quo and ex-ante quantities from consolidating across every health service area m.

This study applies the last formula for other administrative area levels such as sub-district, district, and province across categorical hospital levels such as all hospital levels, within the same hospital levels only, or similar hospital levels.

For standard measurement, this study normalizes the workload per worker to the identical measurement unit of primary-level OP discharge for comparability between outpatient and inpatient services across different hospital levels. This study obtains the national average cost of primary-level OP service from the fitted regression model at 108 Thai Baht. This average cost is multiplied by 0.64 as the national average share of labor cost in the primary hospitals. Consequentially, the total output of all public hospitals in this study is equivalent to the workloads of 1,204,133,398 OP cases at the primary-level hospitals.

Lastly, this study evaluates the economic value of each network consolidation option. The economic value is simply a multiplication of the number of the service delivery units, average workforce per service delivery unit, average OP cases per worker, the average reduction in OP cases per worker, and the average labor cost of the OP case. All these quantities, but the last one, are available from the result tables of area-based network allocation in the main manuscript. The average labor cost of the OP case is calculated from the primary hospitals discussed above.

Therefore, one can multiply the number of provinces with the average workforce per province, average OP cases per worker, average OP case reductions per worker, and the

average labor cost of the OP case to calculate the economic value of the provincial-level consolidation. Similarly, one can conduct such network consolidation calculations of economic valuation for other levels of administrative areas and different categories of hospital levels.

Finally, we can obtain the estimated economic values associated with the network consolidation options such that we can evaluate the appropriate choices which are feasible for the system capabilities and aligned with the system development goals.

Regression results

Table S4: OLS regression of log of cost of outpatient treatments

Dependent variable: log-transformed total cost of outpatient discharge	**Primary**	**First-level secondary**	**Second-level secondary**	**Third-level secondary**	**Tertiary**
Age	-0.0000655***<br>-4.39	0.00756***<br>263.64	0.00897***<br>229.21	0.0110***<br>213.04	0.0115***<br>233.40
Age squared	0.0000249***<br>142.67	-0.0000428***<br>-133.47	-0.0000480***<br>-109.67	-0.0000584***<br>-101.57	-0.0000477***<br>-86.30
Female (relative to male)	-0.0230***<br>-115.30	-0.0105***<br>-29.72	-0.0217***<br>-45.53	-0.0322***<br>-54.73	-0.0550***<br>-95.37
Out-office hours (relative to office hours)	0.00365***<br>14.74	0.0106***<br>29.13	0.0125***<br>25.66	0.0585***<br>96.09	0.0204***<br>34.63
By appointment (relative to walk-in)	0.132***<br>244.82	0.202***<br>439.86	0.302***<br>516.88	0.322***<br>490.24	0.264***<br>423.79
Refer from other hospital (relative to walk-in)	0.187***<br>43.44	0.395***<br>119.61	0.115***<br>31.32	0.347***<br>211.81	0.380***<br>238.65
Refer from emergency service or EMS (relative to walk-in)	-0.0117*<br>-2.49	0.149***<br>22.53	0.273***<br>37.73	-0.0765***<br>-7.74	-0.325***<br>-51.58
CSMBS (relative to UCS)	0.0576***<br>135.21	0.207***<br>390.80	0.205***<br>290.54	0.297***<br>387.17	0.248***<br>320.63

Dependent variable: log-transformed total cost of outpatient discharge	**Primary**	**First-level secondary**	**Second-level secondary**	**Third-level secondary**	**Tertiary**
SSS (relative to UCS)	0.0268***	-0.0771***	-0.0642***	-0.0117***	-0.162***
	65.54	-108.46	-69.03	-12.55	-185.09
OOPE (relative to UCS)	0.0178***	-0.00994***	0.0386***	0.0608***	-0.0530***
	25.71	-12.05	38.20	48.89	-50.82
Health Service Area: 2 (relative to HSA 1)	0.190***	0.191***	0.197***	0.156***	1.495***
	354.45	230.32	145.58	109.72	870.94
Health Service Area: 3 (relative to HSA 1)	-0.00332***	-0.0173***	-0.136***	-0.271***	0.272***
	-6.64	-22.78	-69.90	-185.49	134.61
Health Service Area: 4 (relative to HSA 1)	-0.0589***	0.138***	0.0190***	-0.0232***	0.226***
	-121.33	149.80	17.75	-18.39	149.75
Health Service Area: 5 (relative to HSA 1)	-0.168***	0.125***	0.147***	0.0548***	0.164***
	-357.38	158.06	140.17	43.14	138.35
Health Service Area: 6 (relative to HSA 1)	0.0952***	0.137***	0.0797***	-0.439***	0.642***
	201.35	157.04	75.33	-210.32	557.00
Health Service Area: 7 (relative to HSA 1)	0.176***	0.125***	0.321***	0.0890***	0.271***
	416.72	176.06	269.12	57.45	196.03
Health Service Area: 8 (relative to HSA 1)	0.298***	-0.0218***	0.263***	-0.0597***	0.746***
	730.10	-29.41	187.89	-47.67	447.65
Health Service Area: 9 (relative to HSA 1)	0.0898***	0.0594***	0.113***	-0.419***	0.551***
	219.92	72.63	116.41	-201.59	443.56

Dependent variable:<br>log-transformed total cost of outpatient discharge	**Primary**	**First-level secondary**	**Second-level secondary**	**Third-level secondary**	**Tertiary**
Health Service Area: 10 (relative to HSA 1)	0.0438***<br>90.73	0.713***<br>974.78	0.311***<br>240.47	0.140***<br>99.82	1.128***<br>814.82
Health Service Area: 11 (relative to HSA 1)	0.0134***<br>24.37	0.171***<br>230.21	0.163***<br>138.53	0.125***<br>72.68	0.545***<br>425.76
Health Service Area: 12 (relative to HSA 1)	0.0452***<br>87.40	0.0959***<br>129.37	0.133***<br>112.24	0.0257***<br>20.48	0.131***<br>103.86
Constant	3.744***<br>1120.16	4.814***<br>1612.09	4.659***<br>1171.51	5.076***<br>1148.17	4.645***<br>1109.25
Observations	116,382,110	53,351,160	29,338,847	22,611,176	30,149,270
R-squared	0.158	0.190	0.193	0.182	0.182

Note: The dummy variables for principle diagnostic codes of 140 disease categories are not shown.

* *P*<0.05, ** *P* <0.01, *** *P* <0.001 with *t*-statistics in the second row.

Table S5: OLS regression of log of cost of inpatient treatments

Dependent variable: log-transformed total cost of inpatient discharge	**Primary**	**First-level secondary**	**Second-level secondary**	**Third-level secondary**	**Tertiary**
Age	0.00361<br>1.91	0.00217***<br>14.63	0.00552***<br>36.74	0.0141***<br>66.54	0.0150***<br>95.29
Age squared	-0.0000294<br>-1.45	-0.000000992<br>-0.62	-0.0000148***<br>-9.03	-0.0000909***<br>-39.25	-0.000135***<br>-79.57
Female (relative to male)	-0.0972***<br>-3.49	-0.0393***<br>-19.51	-0.0609***<br>-29.49	-0.0890***<br>-32.01	-0.0691***<br>-34.06
Out-office hours (relative to office hours)	-0.108***<br>-3.76	-0.0387***<br>-19.69	-0.0404***<br>-20.13	-0.334***<br>-125.92	-0.133***<br>-68.67
By appointment (relative to walk-in)	0.172**<br>2.98	-0.182***<br>-47.04	0.0632***<br>18.31	-0.188***<br>-43.79	0.230***<br>83.63
Refer from other hospital (relative to walk-in)	-0.537***<br>-5.85	-0.0334***<br>-4.20	-0.131***<br>-23.18	0.364***<br>102.43	0.867***<br>348.80
Refer from emergency service or EMS (relative to walk-in)	0.315***<br>5.34	-0.0698***<br>-9.01	0.0682***<br>7.85	-0.232***<br>-20.39	0.394***<br>55.34
CSMBS (relative to UCS)	0.326***<br>6.17	0.0485***<br>13.43	-0.00319<br>-0.92	0.235***<br>54.17	0.160***<br>51.09
SSS (relative to UCS)	0.312***	0.0311***	0.0567***	0.209***	-0.0777***

Dependent variable:<br>log-transformed total cost of inpatient discharge	**Primary**	**First-level secondary**	**Second-level secondary**	**Third-level secondary**	**Tertiary**
	4.17	6.01	11.67	36.54	-20.47
OOPE (relative to UCS)	-0.0959	0.280***	0.129***	0.241***	-0.756***
	-0.88	49.12	26.71	41.38	-214.33
Days admitted	0.00747	0.0475***	0.00925***	0.0160***	0.0479***
	0.67	89.77	24.54	60.34	163.15
Days admitted squared	0.000107	-0.000253***	-0.0000822***	-0.0000714***	-0.000172***
	0.16	-23.17	-11.04	-26.99	-52.47
Health Service Area: 2 (relative to HSA 1)		1.276***	-0.0348***	0.335***	1.456***
		198.17	-6.57	45.08	306.84
Health Service Area: 3 (relative to HSA 1)		-0.0558***	1.981***	-2.325***	-3.165***
		-13.87	216.35	-418.11	-998.85
Health Service Area: 4 (relative to HSA 1)		0.0164***	0.111***	-0.823***	0.612***
		3.65	23.51	-124.08	98.87
Health Service Area: 5 (relative to HSA 1)		-0.0519***	0.168***	-0.556***	-2.157***
		-11.23	37.70	-82.30	-669.38
Health Service Area: 6 (relative to HSA 1)	-0.587***	0.687***	0.0298***	-1.938***	-0.304***
	-13.60	106.10	6.72	-306.22	-75.98
Health Service Area: 7 (relative to HSA 1)		-0.0715***	0.341***	-1.218***	-1.929***
		-19.40	68.57	-176.73	-537.22

Dependent variable:<br>log-transformed total cost of inpatient discharge	**Primary**	**First-level secondary**	**Second-level secondary**	**Third-level secondary**	**Tertiary**
Health Service Area: 8 (relative to HSA 1)	-1.004***<br>-22.44	0.126***<br>-13.87	0.434***<br>216.35	-1.297***<br>-418.11	-0.572***<br>-998.85
Health Service Area: 9 (relative to HSA 1)		-0.0432***<br>-10.01	0.306***<br>79.99	-1.838***<br>-215.43	-1.210***<br>-399.01
Health Service Area: 10 (relative to HSA 1)		2.245***<br>520.69	-0.419***<br>-103.04	-2.462***<br>-407.56	1.188***<br>284.75
Health Service Area: 11 (relative to HSA 1)	-1.312<br>-1.19	0.620***<br>3.65	0.258***<br>23.51	0.818***<br>-124.08	-0.756***<br>98.87
Health Service Area: 12 (relative to HSA 1)		-0.0402***<br>-9.35	0.515***<br>96.25	-0.438***<br>-62.97	-2.557***<br>-655.52
Constant	4.254***<br>14.74	4.401***<br>-11.23	4.246***<br>37.70	5.793***<br>-82.30	6.284***<br>-669.38
Observations	4,366	3,006,332	1,886,323	2,250,352	4,456,539
R-squared	0.172	0.214	0.099	0.195	0.331

Note: The dummy variables for principle diagnostic codes of 140 disease categories are not shown.

* *P*<0.05, ** *P* <0.01, *** *P* <0.001 with *t*-statistics in the second row.
